# Supplementary material for: International Approaches to Management of CFTR-Related Metabolic Syndrome/Cystic Fibrosis Screen Positive, Inconclusive Diagnosis
Source: Int J Neonatal Screen. 2022 Jan 11;8(1):5. doi: 10.3390/ijns8010005 (PMC8788507; doi:10.3390/ijns8010005)
Supplement: Supplementary file 1 [file IJNS-08-00005-s001.zip › IJNS-1515116-supplementary.pdf]

CRMS/CFSPID Questionnaire

There are 3 sections to the survey: Section 1: Collects demographic details; Section 2: Asks about the initial assessment; Section 3: Asks about ongoing management

Start of Block: DEMOGRAPHIC DATA

Section 1: This section will include demographic questions

Q1 In which country do you currently work?

Q2 Please can you specify the state or region you work within in this country?

____

Q3 What best describes your current job title?

- ○ Doctor
- ○ Nurse
- ○ Laboratory staff
- ○ Genetic Counsellor
- ○ Centre Director
- ○ Professor / Associate Professor
- ○ Research Scientist
- ○ Newborn Screening Coordinator
- ○ Other, please state ____

Q4 What are your professional qualifications?

- ☐ MD
- ☐ PhD
- ☐ MSc
- ☐ BSc
- ☐ RN
- ☐ Other, please state ____

Q5 How many years have you worked with children with cystic fibrosis and their families?

____

Q6 How many years have you worked with children with a designation of CFTR-related metabolic syndrome/cystic fibrosis screen positive, inconclusive diagnosis (CRMS/ CFSPID)?

____

End of Block: DEMOGRAPHIC DATA

Start of Block: Following the initial NBS result

Section 2: These questions relate to your initial assessment following the Newborn Bloodspot Screening (NBS) result

Q7 Please could you give a brief summary of the information you provide when communicating a CRMS/CFSPID NBS result to families during their initial consultation

____

____

____

____

____

Q8 During the initial assessment following the NBS result, which tests would you perform?

- ▢ Clinical assessment (including respiratory, abdominal and nutritional assessment)
- ▢ Sweat test
- ▢ Extended CFTR analysis (if the genotype is incomplete)
- ▢ Collection of a stool sample for measurement of fecal elastase
- ▢ Other(s), please specify ____

End of Block: Following the initial NBS result

Start of Block: Ongoing management

Section 3: Ongoing management

Q9 Currently, there are two categories for children with a designation of CRMS/CFSPID
1. Normal sweat chloride (< 30mmol/L) and two CFTR mutations, at least one of which has unclear phenotypic consequences
2. Intermediate sweat chloride value (30-59 mmol/L) and one or no CFTR mutations

Do you manage these children differently?

- Yes
- No

Skip To:Q If Currently, there are two categories for children with a designation of CRMS/CFSPID 1. Normal swea... = No

Skip To: Q If Currently, there are two categories for children with a designation of CRMS/CFSPID 1. Normal swea... = Yes

Q10 In what way(s) do you manage the CRMS/CFSPID categories differently?

____

Q11 Do you follow up infants with a CRMS/CFSPID designation in a specialist CF clinic?

- Yes
- No

Skip To: Q If Do you follow up infants with a CRMS/CFSPID designation in a specialist CF clinic? = Yes

Skip To: Q If Do you follow up infants with a CRMS/CFSPID designation in a specialist CF clinic? = No

Q12 Do any policies exist to ensure the infant is not exposed to any increased risk of cross infection?

- Yes
- No

Skip To: Q If Do any policies exist to ensure the infant is not exposed to any increased risk of cross infection? = Yes

Skip To: Q16 If Do any policies exist to ensure the infant is not exposed to any increased risk of cross infection? = No

Q13 Is there any reason why you do not have a policy in place to ensure the infant is not exposed to any increased risk of cross infection?

- ○ We do not feel it is necessary
- ○ We do not have the capacity (time, space or staff)
- ○ Other, please specify ____

Q14 Please could you briefly describe what these policies consist of.

- ▢ Each child and family placed in a separate room
- ▢ Staff are required to wear gloves and apron when with the child and family
- ▢ Staff are required to wash their hands prior to and after the consultation
- ▢ Children with CFSPID are seen in a separate clinic to children with CF
- ▢ Children with CFSPID are seen in the same clinic as children with CF but either at the beginning or end of the clinic
- ▢ Other, please specify ____

Display This Question:

If Do you follow up infants with a CRMS/CFSPID designation in a specialist CF clinic? = No

Q15 Are there any reasons why you do not follow these infants up in a specialist clinic?

- ☐ We do not feel there is any need to see these children in a specialist CF clinic
- ☐ We do not have the capacity (time or staff) to see these children as well as children with CF
- ☐ We think this might confuse parents and make them think their child has CF
- ☐ Other, please specify____

Q16 Do you offer these infants a repeat sweat test at any point

- ◯ Yes
- ◯ No

Skip To: Q If Do you offer these infants a repeat sweat test at any point = Yes

Skip To: Q If Do you offer these infants a repeat sweat test at any point = No

Q17 At what age do you offer the repeat sweat test?

- ◯ 6 months
- ◯ 1 year
- ◯ 18 months
- ◯ 2 years
- ◯ Other, please specify ____

Display This Question:

If Do you offer these infants a repeat sweat test at any point = No

Q18 Is there any reason(s) why you do not offer a repeat sweat test?

- ○ We do not feel it is necessary
- ○ We do not have the resources (time, staff, finances)
- ○ We feel it causes parents unnecessary anxiety
- ○ Other, please state ____

Q19 What clinical or other information (e.g. parental anxiety) would you take into consideration when determining how frequently you review these children?

- ☐ NBS result
- ☐ Sweat Chloride
- ☐ Clinical assessment (including respiratory, abdominal and nutritional assessment)
- ☐ Pulmonary function
- ☐ Chest X-ray
- ☐ Magnetic resonance imaging of chest
- ☐ Chest computerized tomography scan
- ☐ Cough swab / respiratory cultures
- ☐ Parental anxiety
- ☐ Other, please specify ____

Q20 For infants that have no clinical concerns, how frequently would you review them after 12 months of age?

- ○ Annually
- ○ Every two years
- ○ We would not routinely review them
- ○ Other, please state ____

Skip To: Q If For infants that have no clinical concerns, how frequently would you review them after 12 months... = We would not routinely review them

Q21 Please indicate which tests or measurements children would have during or in preparation for their review appointment (if this is always the same, please indicate in the 'we undertake the following' column, if this different depending on the age of the child, please indicate this using the relevant age columns)

	We undertake the following (16)	Aged 1 year (1)	Aged 2 years (2)	Aged 3 years (3)	Aged 4 years (4)	Aged 5 years (5)	Aged 6 years (6)	Aged 7 years (7)	Aged 8 years (8)	Aged 9 years (9)	Aged 10 years (10)	Aged 11 years (11)	Aged 12 years (12)	Aged 13 years (13)	Aged 14 years (14)	Aged 15 years (15)
Full blood count / Complete blood count																
Urea and electrolytes																
Pulmonary function																
Chest X-ray																
Magnetic resonance imaging of chest																
Chest computerized tomography scan																
Bronchoalveolar lavage																

Cough swab / respiratory cultures	☐	☐	☐	☐	☐	☐	☐	☐	☐	☐	☐	☐	☐	☐	☐	☐
Stool sample for fecal elastase	☐	☐	☐	☐	☐	☐	☐	☐	☐	☐	☐	☐	☐	☐	☐	☐
Other, please specify	☐	☐	☐	☐	☐	☐	☐	☐	☐	☐	☐	☐	☐	☐	☐	☐
Other, please specify	☐	☐	☐	☐	☐	☐	☐	☐	☐	☐	☐	☐	☐	☐	☐	☐

Q22 Do you review the CFTR-2 / CFTR-France website prior to the review?

◯ Yes

◯ No

Skip To: Q If Do you review the CFTR-2 / CFTR-France website prior to the review? = Yes
Skip To: Q If Do you review the CFTR-2 / CFTR-France website prior to the review? = No

Q23 Are there any reasons why you do not review the CFTR-2 / CFTR-France website prior to the review?

▢ Difficult to access

▢ Time consuming to access

▢ I do not feel the information is relevant for the purposes of the annual review

▢ I do not feel the information is useful for the purposes of the annual review

▢ Other, please speficy

Q24 Why do you do this and do you find it useful?

____

Q25 Do you do any respiratory cultures at the review appointment or at any other times?

◯ Yes

◯ No

Skip To: Q If Do you do any respiratory cultures at the review appointment or at any other times? = No
Skip To: Q If Do you do any respiratory cultures at the review appointment or at any other times? = Yes

Q26 How frequently do you do respiratory cultures?

- ◯ Monthly
- ◯ Every 2 months
- ◯ Every 3 months
- ◯ Every 6 months
- ◯ Annually
- ◯ Other, please specify ____

Display This Question:

If Do you do any respiratory cultures at the review appointment or at any other times? = No

Q27 Why do you not do respiratory cultures at the review appointment?

- ▢ Too time consuming
- ▢ Do not have the resources (time, staff, finances)
- ▢ Do not feel this information informs the annual review
- ▢ Other, please specify ____

Display This Question:

If For infants that have no clinical concerns, how frequently would you review them after 12 months... = We would not routinely review them

Q28 Are there any reason(s) why you do not offer routine review(s)?

- ☐ We do not have capacity (time, space, staff, finances)
- ☐ We feel it makes families more anxious
- ☐ We do not believe there is any evidence to suggest this is beneficial
- ☐ We do not feel there is any need to see these children routinely
- ☐ other, please specify ____

Q29 What information do you give to families regarding when they should seek additional medical advice about their child?

- ☐ Failure to gain weight adequately
- ☐ Persistent loose stools
- ☐ Persistent respiratory symptoms (more than 2 weeks)
- ☐ Other, please specify ____

Q30 What information do you give to the primary care physician / general practitioner regarding when they should seek additional medical advice about their child?

- ☐ Failure to gain weight adequately
- ☐ Persistent loose stool
- ☐ Persistent respiratory symptoms (more than 2 weeks)
- ☐ Other, please specify ____

Q31 What advice to you give to families regarding their child's immunisations?

○ Children should follow the national immunisation programme

○ Parents should avoid specific immunisations, please state which ones ____

○ Parents should delay specific immunisations, please state which ones ____

○ Other, please state ____

Q32 What health promotion advice do you give to families of these infants?

☐ Children should not be exposed to cigarette smoke

☐ Children and their families should be encouraged to adopt a healthy lifestyle consistent with national guidance on exercise, nutrition and other aspects of public health policy

☐ The family should avoid keeping pets if at all possible

☐ Other, please specify ____

Q33 Do you offer families a referral for genetic counselling

○ Yes

○ No

Skip To: Q If Do you offer families a referral for genetic counselling = Yes

Skip To: Q If Do you offer families a referral for genetic counselling = No

Q34 When do you normally discuss this / make the referral?

- ◯ At the initial consultation with the family
- ◯ During the annual review
- ◯ Other, please specify ____

Q35 How long does it normally take after the referral before the family is seen by the genetic service?

- ◯ 1 week
- ◯ 1 month
- ◯ 2 months
- ◯ 3 months
- ◯ 6 months
- ◯ 1 year
- ◯ Other, please specify ____

Display This Question:

If Do you offer families a referral for genetic counselling = No

Q36 Is there any reason why you do not make a referral for genetic counselling?

- [ ] I do not feel it is necessary
- [ ] Parents have not requested this
- [ ] It is not part of our policy
- [ ] I think this adds to parental anxiety
- [ ] Other, please specify ____

Q37 Is there a national database where the infants' details can be stored?

- ○ Yes, we use the CF-registry
- ○ Yes, we have a separate CRMS/CFSPID-registry
- ○ No

Q38 The updated guidance from the ECFS working group suggests children with CRMS/CFSPID who reach 6 years of age in good health with normal growth, lung function and imaging and normal sweat chloride values (<30 mmol/L) are unlikely to convert to a diagnosis of CF. In this instance, which option for further care do you currently support:

- ○ Discharge from CF specialist care, with follow-up in primary care by PCP.
- ○ Discharge from CF specialist care, but a further isolated specialist review as the child reaches adolescence (at the age of around 14-16 years, see below). This option gives the opportunity for more direct engagement with the young person.
- ○ Continue regular specialist review under the CRMS/CFSPID designation, either as part of the CF clinic or in a separate clinic (could be undertaken 'virtually' for example as an annual telephone call or video consultation).
- ○ Other, please specify ____

Q39 The updated guidance for CRMS/CFSPID from the ECFS working group suggests that for children with CRMS/CFSPID who are discharged from specialist care, a subsequent review should be considered when the child is a young adult, to communicate the information directly to them. Do you think this type of review should be organised?

- ○ Yes
- ○ No

Skip To: Q If The updated guidance for CRMS/CFSPID from the ECFS working group suggests that for children with... = Yes

Skip To: End of Block If The updated guidance for CRMS/CFSPID from the ECFS working group suggests that for children with... = No

---

Q40 At what age do you think the review should take place?

____

End of Block: Ongoing management

Start of Block: Thank you

You have reached the end of the survey. Thank you for taking part in this research. If you would like further information, please contact the research team.

End of Block: Thank you
